# Malakoplakia of the Gallbladder: A Case Report

**DOI:** 10.7759/cureus.38912

**Published:** 2023-05-11

**Authors:** Anita B Sajjanar, Sunita Vagha

**Affiliations:** 1 Pathology, Datta Meghe Medical College, Nagpur, IND; 2 Pathology, Jawaharlal Nehru Medical College, Datta Meghe Institute of Higher Education and Research, Wardha, IND

**Keywords:** gallbladder, pas stain, histopathology, malakoplakia, histiocytes

## Abstract

Histiocytes are cells that are involved in the immune responses of the body. They are unable to properly break down the bacterial material in malakoplakia, a chronic granulomatous histiocytic disease that occurs in immunocompromised patients and autoimmune conditions. Very few reports of these lesions exist, as those that occur in the gallbladder. It typically affects the urinary bladder, alimentary tract, cutaneous, hepato-biliary, and male and female genital systems. These lesions are usually incidental findings that result in patients being misdiagnosed.

A 70-year-old female presented with right lower quadrant abdominal pain, and malakoplakia of the gallbladder was diagnosed. Histopathology findings revealed malakoplakia of the gallbladder, and the same was confirmed with special stains such as periodic acid-Schiff (PAS). This case highlights the role of gross and histopathology findings as a clue to the diagnosis, which helps the surgeon with further management.

## Introduction

A condition called malakoplakia is characterized by chronic granulomatous inflammation of various organs, including the genitourinary system [1], but in addition, the respiratory system, gastrointestinal tract, central nervous system, skin, and hepato-biliary system are affected [2]. The etiopathogenesis has not been understood completely, but a few theories that have been suggested include the role of microorganisms particularly *Escherichia coli*, *Mycobacterium tuberculosis*, and *Staphylococcus aureus*, which may be causative [3], and an abnormally altered immune response and the defect of lysosomal function lead to decreased macrophage response. In malakoplakia, it has been suggested that macrophages are capable of phagocytosis but unable to digest and clear the bacteria.

Xanthogranulomatous cholecystitis and malakoplakia can present in a similar fashion and are thought to be part of the spectrum of chronic inflammatory pathology, with the difference of malakoplakia being more aggressive [4].

Malakoplakia arising from the gallbladder is reported in the literature in very few cases. It is an incidental finding, and frequently, patients are misdiagnosed clinically as having a malignant condition. A 70-year-old female with chronic cholecystitis and gallstone cholecystolithiasis presented with malakoplakia. The gallbladder was removed via laparoscopic surgery. Later histopathology findings reported malakoplakia of the gallbladder, and this was confirmed with special stains such as periodic acid-Schiff (PAS). This case highlights the role of gross and histopathology findings as clues to the diagnosis, which helps the surgeon with further management.

## Case presentation

A 70-year-old female complained of pain in the abdomen, right upper quadrant, for one month, which was clinically suspected as chronic calculus cholecystitis. Her physical and abdominal examination was normal.

**Figure 1:**
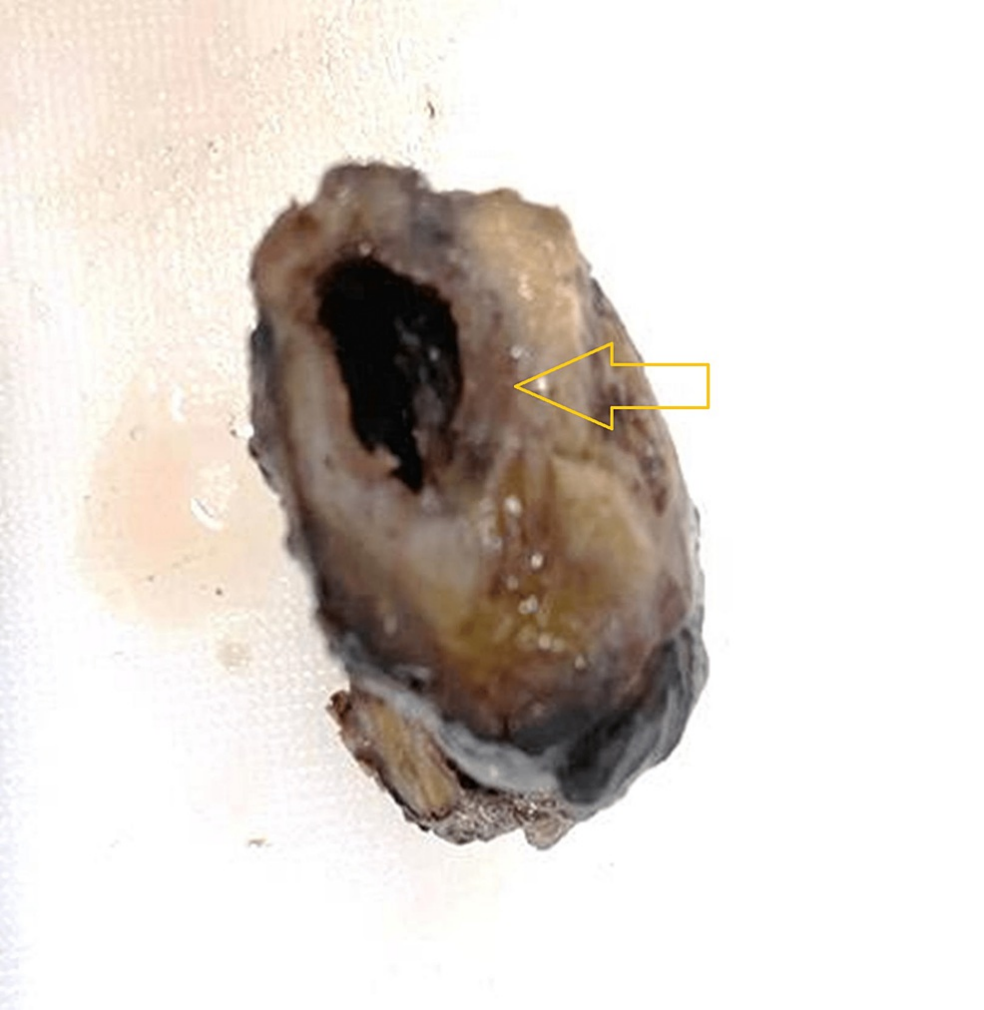
Partially cut-opened gallbladder with an irregular and congested external surface (yellow arrow)

**Figure 2:**
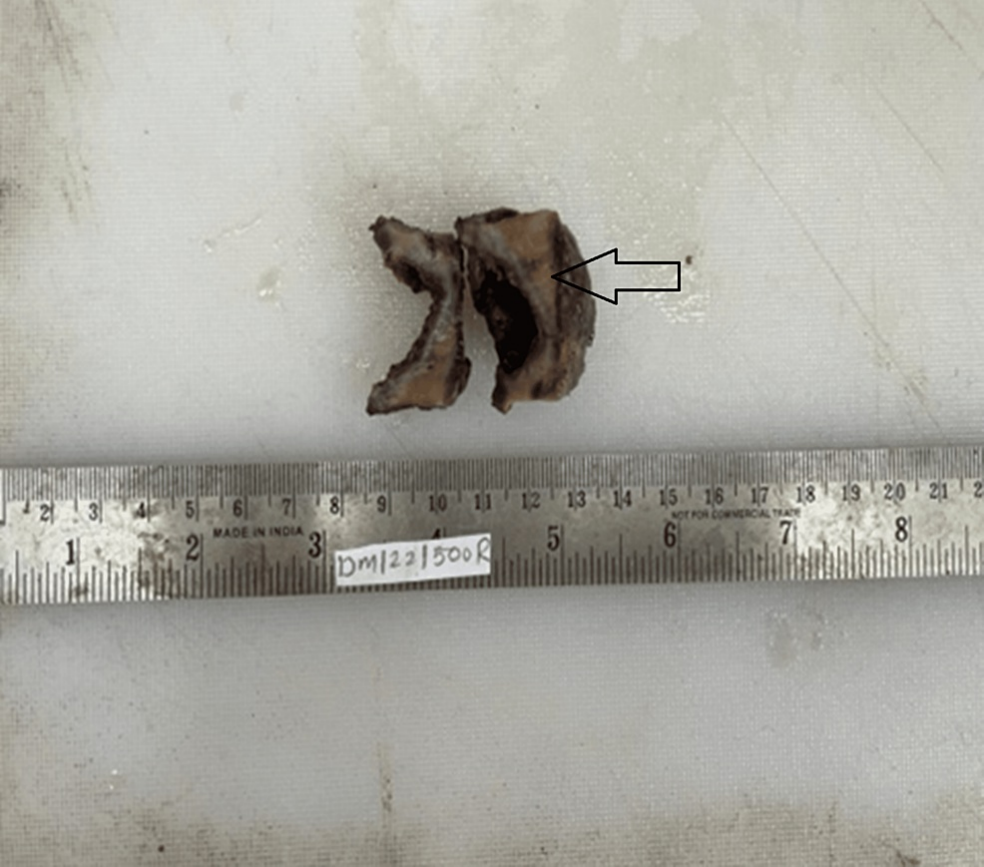
Cut surface showing gray-brown to yellowish areas with thin mucosa (black arrow)

Ultrasonography (USG) of the abdomen and contrast-enhanced computed tomogram (CECT) of the abdomen showed a distended gallbladder, multiple calculi were noted within the neck region, and the wall appears irregular. The patient underwent laparoscopic cholecystectomy. The patient is on follow-up and is symptom-free. A cut-opened gallbladder measuring 5 cm long with an irregular and congested external surface was shown on gross examination. The cut surface shows a gray-brown area with thin mucosa, and tiny mixed stones were seen (Figures 1-2).

Sections studied from the gallbladder show mucosa lined by columnar epithelium, Rokitansky sinus, and lymphocytic infiltration in the lamina propria. The fibrosis and thickening of muscularis propria were noted.

**Figure 3:**
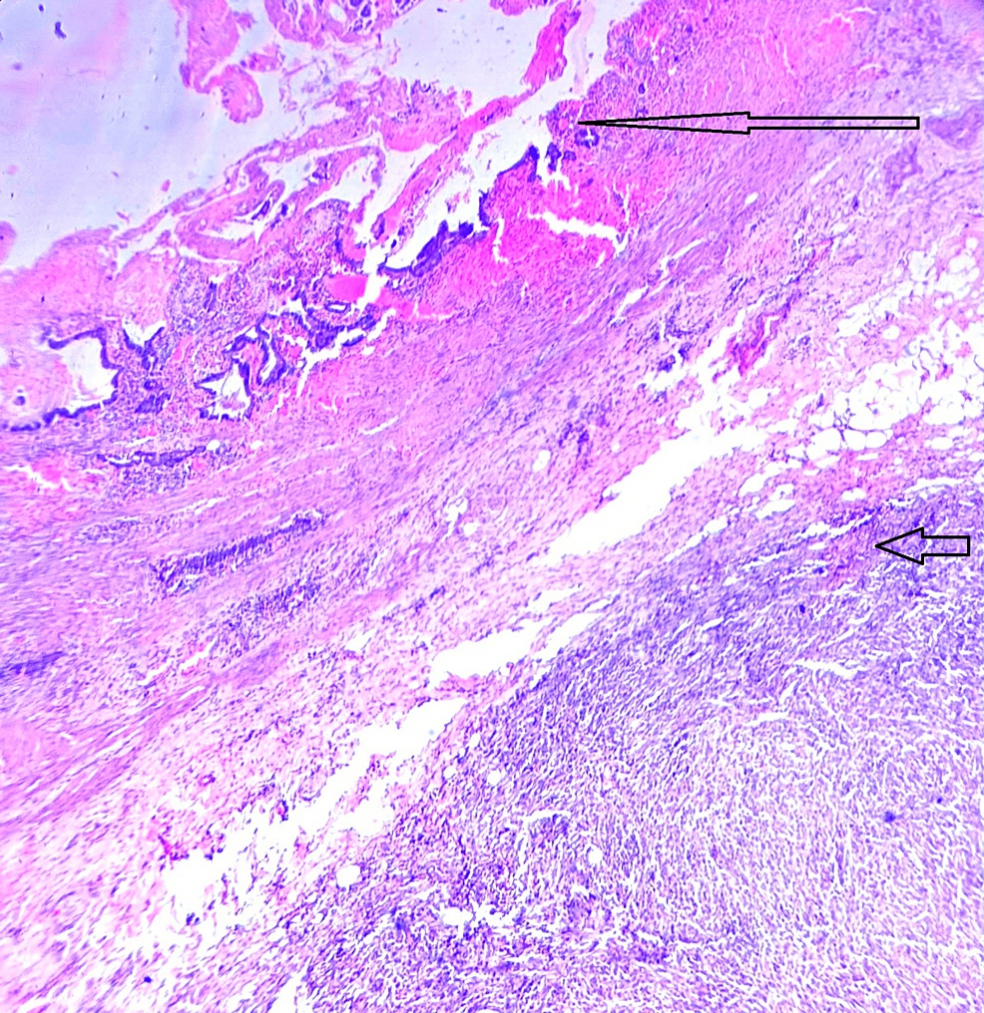
Low-power view of the gallbladder showing mucosa (thin black arrow) and the serosa showing the collection of histiocytic granuloma (thick black arrow)

**Figure 4:**
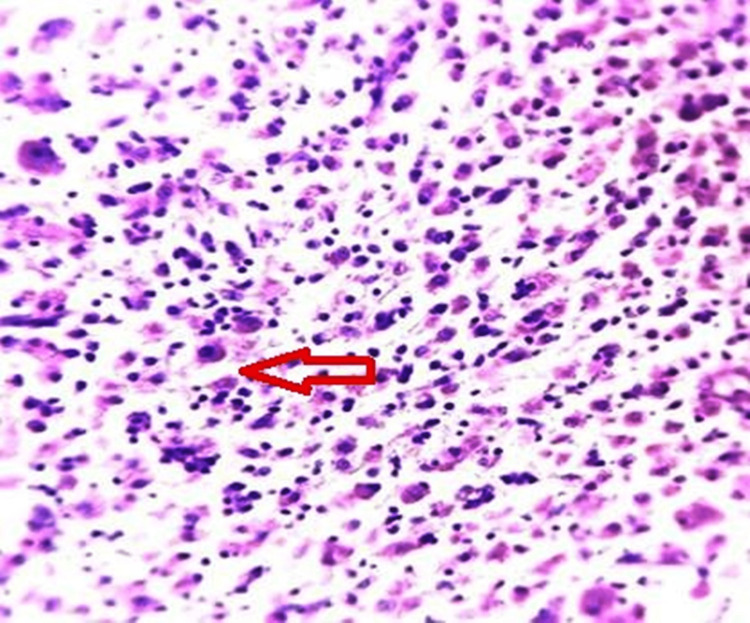
Confluent sheets of histiocytes with eosinophilic granular cytoplasm and eccentric nuclei (H&E 10×) (red arrow)

The serosa shows few granulomas comprising confluent sheets of histiocytes with eosinophilic granular cytoplasm and eccentric nuclei. Occasional Michaelis-Gutmann bodies were seen, and the microscopic examination reported a malakoplakia of the gallbladder (Figures 3-4).

**Figure 5:**
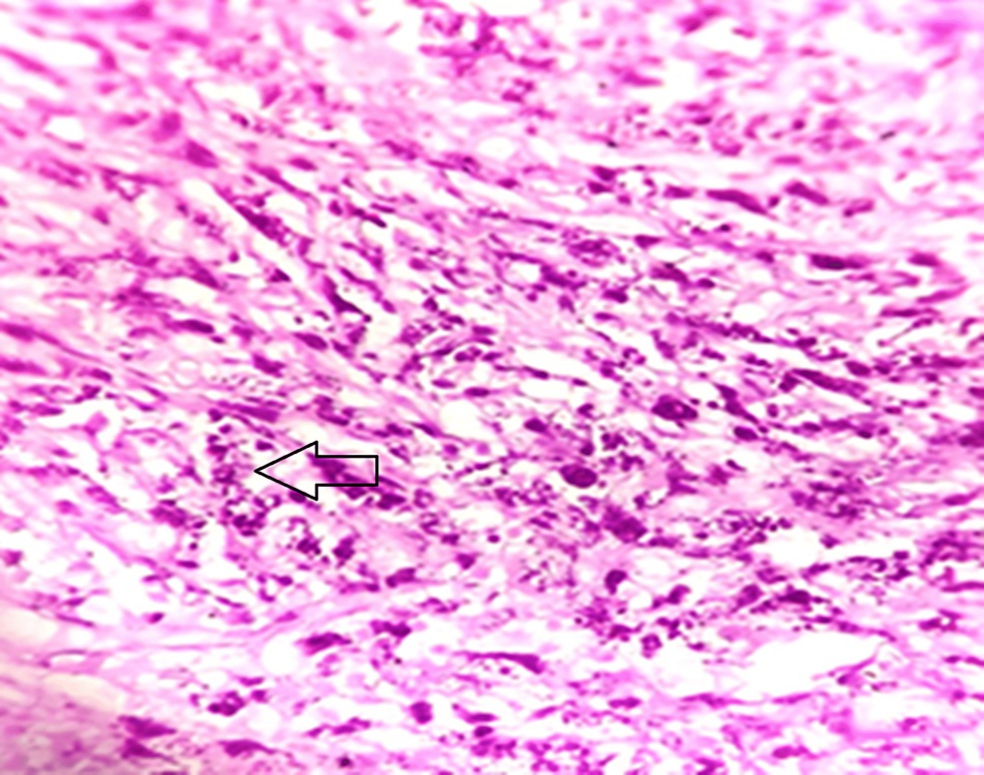
Periodic acid-Schiff stain for granular cytoplasm (black arrow)

Later, malakoplakia of the gallbladder was confirmed with special stains such as periodic acid-Schiff (PAS) (Figure 5).

## Discussion

Malakoplakia was first described by Michaelis and Gutmann in 1902 and later named by von Hansemann. The term “malakoplakia” is composed of the Greek words malakos (soft) and plakos (plaque) [5]. An abnormal immune response, defective lysosome activity resulting in reduced cyclic guanosine monophosphate to kill bacteria, or a bacteria-specific process is believed to be responsible for this condition [6]. Bacteria are phagocytized by macrophages, but partially digested bacterial components accumulate within the phagolysosome [7].

Ten cases of malakoplakia of the gallbladder have been reported in the literature, as described by Hanada et al. [7]. This study observed that gross lesions appear as soft, flat, yellowish, slightly raised plaques or nodules and vary in diameter from 0.5 to 5 cm. Microscopically, plaques are composed of sheets of foamy macrophages with granular cytoplasm, which are known as von Hansemann cells. Michaelis-Gutman bodies are pathognomonic of this disorder, ultrastructurally representing lysosomes filled with partly digested debris of bacteria by phagosome due to defective phagocytosis. These bodies are round to oval basophilic inclusion (cytoplasmic laminated concretions of calcium phosphate). Special stains such as periodic acid-Schiff stain for granular cytoplasm and von Kossa’s stain for calcium and iron are used to confirm the diagnosis [8].

Pathologists’ identification of malakoplakia is critical in deciding on the proper treatment for the patient because antibiotic therapy (trimethoprim-sulfamethoxazole, rifampicin, and quinolones) is both effective and curative. Bethanechol chloride is a cholinergic agonist that increases cyclic guanosine levels, thereby enhancing the lysosomal bactericidal activity in macrophages that have been impaired [9].

A further case of malakoplakia of the gallbladder arising from xanthogranulomatous cholecystitis and associated with infection is described by Di Tommaso et al. This case explained that an impaired phagocytic process is thought to be the cause of xanthogranulomatous cholecystitis and malakoplakia of the gallbladder, which has a similar clinical picture. However, the histological features may vary considerably. Thus, they concluded that the cases of cholecystitis caused by malakoplakia may be difficult to diagnose and may be confused with xanthogranulomatous cholecystitis and gallbladder malignancy [10].

## Conclusions

Malakoplakia of the gallbladder is a diagnostic dilemma faced by pathologists, radiologists, and surgeons. It is a rare histiocytic disorder and is often misdiagnosed as malignancy clinically. Also, one should be aware of differentiating xanthogranulomatous cholecystitis from malakoplakia histopathologically as treatment modalities vary. Here, we have highlighted the rare case of malakoplakia of the gallbladder. Gross and histopathology findings are clues to the diagnosis.
